# Effects of dietary *Clostridium butyricum* and rumen protected fat on meat quality, oxidative stability, and chemical composition of finishing goats

**DOI:** 10.1186/s40104-023-00972-8

**Published:** 2024-01-15

**Authors:** Meimei Zhang, Zhiyue Zhang, Xinlong Zhang, Changming Lu, Wenzhu Yang, Xiaolai Xie, Hangshu Xin, Xiaotan Lu, Mingbo Ni, Xinyue Yang, Xiaoyang Lv, Peixin Jiao

**Affiliations:** 1 College of Animal Science and Technology, Northeast Agricultural University, Harbin, 150030 People’s Republic of China; 2 Lethbridge Research and Development Centre, Lethbridge, T1J 4B1 Canada; 3 School of Agriculture and Food Sciences, The University of Queensland, Gatton, QLD 4343 Australia; 4 International Joint Research Laboratory in Universities of Jiangsu Province of China for Domestic Animal Germplasm Resources and Genetic Improvement, Yangzhou, 225009 China

**Keywords:** Chemical composition, *Clostridium butyricum*, Goats, Meat quality, Oxidative stability, Rumen protected fat

## Abstract

**Background:**

*Clostridium butyricum* (CB) is a probiotic that can regulate intestinal microbial composition and improve meat quality. Rumen protected fat (RPF) has been shown to increase the dietary energy density and provide essential fatty acids. However, it is still unknown whether dietary supplementation with CB and RPF exerts beneficial effects on growth performance and nutritional value of goat meat. This study aimed to investigate the effects of dietary CB and RPF supplementation on growth performance, meat quality, oxidative stability, and meat nutritional value of finishing goats. Thirty-two goats (initial body weight, 20.5 ± 0.82 kg) were used in a completely randomized block design with a 2 RPF supplementation (0 vs. 30 g/d) × 2 CB supplementation (0 vs. 1.0 g/d) factorial treatment arrangement. The experiment included a 14-d adaptation and 70-d data and sample collection period. The goats were fed a diet consisted of 400 g/kg peanut seedling and 600 g/kg corn-based concentrate (dry matter basis).

**Result:**

Interaction between CB and RPF was rarely observed on the variables measured, except that shear force was reduced (*P* < 0.05) by adding CB or RPF alone or their combination; the increased intramuscular fat (IMF) content with adding RPF was more pronounced (*P* < 0.05) with CB than without CB addition. The pH_24h_ (*P* = 0.009), a* values (*P* = 0.007), total antioxidant capacity (*P* = 0.050), glutathione peroxidase activities (*P* = 0.006), concentrations of 18:3 (*P* < 0.001), 20:5 (*P* = 0.003) and total polyunsaturated fatty acids (*P* = 0.048) were increased, whereas the L* values (*P* < 0.001), shear force (*P* = 0.050) and malondialdehyde content (*P* = 0.044) were decreased by adding CB. Furthermore, CB supplementation increased essential amino acid (*P* = 0.027), flavor amino acid (*P* = 0.010) and total amino acid contents (*P* = 0.024) as well as upregulated the expression of lipoprotein lipase (*P* = 0.034) and peroxisome proliferator-activated receptor γ (*PPARγ*) (*P* = 0.012), and downregulated the expression of stearoyl-CoA desaturase (*SCD*) (*P* = 0.034). The RPF supplementation increased dry matter intake (*P* = 0.005), averaged daily gain (trend, *P* = 0.058), hot carcass weight (*P* = 0.046), backfat thickness (*P* = 0.006), concentrations of 16:0 (*P* < 0.001) and *c*9-18:1 (*P* = 0.002), and decreased the shear force (*P* < 0.001), isoleucine (*P* = 0.049) and lysine content (*P* = 0.003) of meat. In addition, the expressions of acetyl-CoA carboxylase (*P* = 0.003), fatty acid synthase (*P* = 0.038), *SCD* (*P* < 0.001) and *PPARγ* (*P* = 0.022) were upregulated due to RPF supplementation, resulting in higher (*P* < 0.001) content of IMF.

**Conclusions:**

CB and RPF could be fed to goats for improving the growth performance, carcass traits and meat quality, and promote fat deposition by upregulating the expression of lipogenic genes of *Longissimus thoracis* muscle.

## Background

In the past few decades, China has turned from a low-income country into a mid-income country with near one-fifth of the world's population. The increased income leads to not only promote the total meat consumption, but also increase the demand for high-quality meat with high nutritional content and sensory properties [1, 2]. Mutton is considered to be a highly nutritious and valuable food because it is rich in high biological value proteins, low cholesterol and rich vitamins [3]. Therefore, during the fattening period of goats, it is not only the feed efficiency and growth rate that need to be pursued, but also the dietary impact on the carcass and meat quality should be considered. Probiotics are regarded as potential alternatives to antibiotics to regulate gastrointestinal microecological balance and improve growth performance and meat quality [4, 5]. *Clostridium butyricum* (CB), a Gram-positive bacterium, has been reported to be a potent feed additive to improve growth performance, feed conversion efficiency and antioxidant capability in monogastric [6]. Previous studies have been focused on its role in adjusting the intestinal microflora structure because CB can survive at low stomach pH and high bile concentrations [7]. To date, CB has been widely used as an alternative to antibiotics in the improvement of growth performance and health of animals, especially in poultry [8] or pigs [9]. However, there is still limited research on the effects of CB on growth performance, and the results are inconsistent in ruminants [10, 11]. In addition, dietary CB can also improve the meat quality of broilers and pigs, which may be related to regulating nutrient digestibility, improving muscle amino acid (AA) and fatty acid (FA) profiles, and enhancing antioxidant status [12, 13]. Moreover, butyric acid produced by CB metabolism can regulate muscle lipid metabolism [14]. Furthermore, the inconclusive effects of CB in the literature may be due to differences in source of CB strains used, its supplemental dose and type of diet [15, 16]. Zhang et al. [17] observed an interaction between dietary lipids and CB on lipid-related gene expression in breast muscle of broiler chickens, suggesting that dietary fat may be interacted with CB on meat quality. The information is scarce on the effects of dietary CB supplementation on meat quality and FA composition of ruminants fed diets varying with fat contents.

The close association of intramuscular fat (IMF) with meat quality, tenderness and flavor as well as the water holding capacity of meat is well documented [18]. Studies reported that dietary fat supplementation to ruminants improved animal productivity by increasing the dietary energy density, providing essential FA and increasing IMF concentration [19, 20]. The FA composition of meat in ruminants is largely influenced by dietary composition, thus the efforts have been made to alter the FA composition and content of animal tissues by inclusion of dietary fat. Therefore, we hypothesized that dietary supplementation with CB and rumen protected fat (RPF) could improve meat quality, nutritional value and promote fat deposition of muscle, and their effects would be interacted. The objective of this study was to investigate the effects of dietary supplementation with CB and RPF on carcass traits, meat quality, muscular antioxidant capacity, oxidative stability, AA and FA composition, and lipid metabolism of *Longissimus thoracis* (LT) muscle in goats.

## Methods

### Animals, experimental design and treatments

Thirty-two male Saanen goats with an average age of 3 months and an initial body weight (BW) of 20.5 ± 0.82 kg were used in the study. The experiment was a completely randomized block design with a 2 × 2 factorial treatment arrangement: 2 RPF supplementation (0 vs. 30 g/d) were combined with 2 CB supplementation (0 vs. 1.0 g/d). The goats were blocked by BW and allocated into 8 blocks of 4 goats. Within each block, goats were randomly assigned to one of the 4 treatments. The product of RPF consists of 48% C16:0, 5% C18:0, 36% C18:1, 9% C18:2 and 2% C14:0, and was provided by Yihai Kerry Food Industry Co., Ltd. (Tianjin, China). The product of CB (2 × 10^8^ CFU/g) was provided from Greensnow Biological Biotechnology Co., Ltd. (Wuhan, China). The dose of the CB was determined based on the manufacturer’s recommendation as well as the previous studies [10, 11], while the dose of RPF was determined according to the report by Behan et al. [21]. The goats were housed individually with free access to water and provided ad libitum a total mixed ration (TMR) twice daily at 08:00 and 18:00. The daily dose of CB and RPF was mixed with 10 g of ground corn, and were top dressed onto the ration twice in the morning and the afternoon feeding. The ingredients and composition of the experimental diets are shown in Table 1. The whole experiment period was consisted of a14-d for adaptation and a 70-d for data and sample collection.

**Table 1:** Ingredients and chemical composition of experimental diets

Item^a^	Content
Ingredients, g/kg dry matter (DM)	
Peanut seedling	400.0
Ground corn	307.0
Soybean meal	76.8
Rice bran meal	42.2
DDGS	36.0
Corn germ meal	24.0
Corn husk	84.0
Gelatinized urea	6.0
Salt	6.0
Premix^b^	18.0
Chemical composition, g/kg DM	
DM, g/kg	913.4
CP	142.3
EE	24.3
NDF	456.9
ADF	212.5
Calcium	9.1
Phosphorous	5.7
Metabolizable energy^c^, MJ/kg	9.21

^a^*CP* Crude protein, *EE* Ether extract, *NDF* Neutral detergent fiber, *ADF* Acid detergent fiber

^b^Supplied per kilogram of dietary DM: vitamin A, 16,000 IU; vitamin D_3_, 1,400 IU; vitamin E, 100 IU; Cu, 8.5 mg; Fe, 50 mg; Zn, 45 mg; Mn, 27 mg; I, 0.5 mg; Se, 0.5 mg; Co, 0.4 mg

^c^Metabolizable energy was a calculated value

### Data and sample collection

#### Feed intake and growth performance

Feed offered and refusals of each goat were recorded daily during the sample collection period to calculate the dry matter intake (DMI). The BW of each goat was recorded at the beginning and the end of collection period before the morning feeding to determine the average daily gain (ADG) and feed conversion ratio (FCR).

#### Slaughtering, carcass traits and sample collection

The goats were weighed as the live weight before slaughter (LWBS) after 16 h of fasting from solid food on the second day after the end of the experiment and slaughtered in a commercial abattoir (Changhao, Harbin, China) in the early morning. After removing the hairs, head, viscera and hoofs, the hot carcass weight (HCW) was recorded and dressing percentage was calculated individually as HCW divided by LWBS × 100. The liver, heart, spleen and kidneys were weighed and organ index was calculated as percentages of live weight. Back fat thickness was measured at the midpoint of the LT muscle at the 12^th^ and 13^th^ rib. A section of LT muscle from the right side of each carcass were frozen at −20 °C for chemical composition, AA and FA analysis. Another LT section was frozen in liquid nitrogen for antioxidant status and gene expression determination.

### Laboratory analysis

#### Chemical analysis

The chemical composition of DM (No. 930.15), crude protein (CP, No. 984.13) and ether extract (No. 920.39) of the feed ingredients, diets and LT muscle were analyzed according to the Association of Analytical Chemists method (AOAC) [22]. Contents of heat-stable α-amylase treated neutral detergent fiber (NDF) was analyzed following the methods of Van Soest et al. [23], and acid detergent fiber (ADF, No. 973.18) was determined according to AOAC [22].

#### Meat quality measurement

The pH value of the LT muscle at 45 min and 24 h (stored in air at 4 °C for 24 h) after slaughtering was determined by inserting a portable pH meter (HI9125; Hanna Instruments, Padova, Italy) with temperature compensation to probe directly into the muscle. The pH meter was calibrated at two points with two kinds of standard buffers (pH = 6.86 and 4.01) before measurement. The meat color parameters including L* (lightness), a* (redness), and b* (yellowness) were determined using a portable chromameter (CR-400, Minolta, Osaka, Japan) under a D65 light source, with a 10^◦^ standard observer, an 8 mm diameter measuring area and a 50 mm diameter illumination area (meat sample was stored in a vacuum bag, taken out before measurement and allowed to bloom for 30 min at 4 °C). Approximately 12 g of meat sample was trimmed to regular pieces (2 cm × 2 cm × 2 cm) and initially weighed. Then, the sample suspended at 4 °C for 24 h and blotted dry on filter paper and reweighed. The drip loss was calculated as the difference before and after drip to the percentage of original weight. The cooking loss and shear force of 32 goat muscle samples were measured simultaneously. Cooking loss was assessed as difference before and after cooking. Briefly, approximately 25 g of meat sample (4 cm × 2 cm × 2 cm) was weighed, wrapped in sealed bags, and heated in a water bath until the central inner temperature reached 70 °C. After cooling and drying at room temperature, the cooked sample was reweighed. Following the cooking loss determination, the same meat samples were used to evaluate the shear force according to the method of Garba et al. [24]. The meat sample was cut into a cuboid of 1 cm × 1 cm × 2 cm along the direction of the muscle fiber and then cut perpendicular to the muscle fiber by a tenderization analyses (C-LM3B tenderization instruments, Northeast Agricultural University, Harbin, China) with a 15-kg load transducer, a crosshead speed of 200 mm/min, and a shearing action similar to a Warner–Bratzler shear device. The samples were cut parallel to the longitudinal orientation of the myofibers. Each sample was measured 6 times, and the average was calculated.

#### Muscle antioxidative status

The activities of total antioxidant capacity (T-AOC, No. A015), superoxide dismutase (SOD, No. A001), glutathione peroxidase (GPX, No. A005), catalase (CAT, No. A007), and the contents of malondialdehyde (MDA, No. A003) in muscle were determined using assay kits according to the manufacturer's instructions (Nanjing Jiancheng Bioengineering Institute, Nanjing, China).

#### Amino acid analysis

The AA profiles of LT muscle were analyzed using the standard method GB 5009.124–2016 [25]. The freeze-dried muscle samples (1.5 g) was added with 10 mL of 6 mol/L HCl solution, and hydrolyzed at 110 °C for 22 h after filling with nitrogen. The solutions were centrifuged, precipitated and dried using an evaporator. Then, the obtained residue was dissolved by adding 2 mL of sodium citrate buffer solution, and was analysed for AA composition using an automatic amino acid analyzer (LA 8080, HITACHI, Tokyo, Japan) after filtered through a 0.22-μm membrane.

#### Fatty acid analysis

Lipids of the freeze-dried muscle and the feed samples were extracted using a mixture of chloroform–methanol (2:1, v/v) according to the proceduces of Folch et al. [26]. Total FA from LT muscle were transesterified into FA methyl esters (FAME) with boron trifluoride-methanol solution reagent, according to He et al. [27]. The FAME were analyzed using an Agilent 6890N gas chromatography equipped with a flame ionization detector (Agilent Technologies) and a CD-2560 (100 m × 0.25 mm × 0.20 µm) capillary column. The gas chromatography program had initial temperature at 75 °C for 30 s, then increased to 175 °C at the rate of 20 °C/min, held for 25 min, increased again from 175 to 215 °C at 10 °C/min and finally held at 215 °C for 40 min. The injector and detector temperature were 235 °C and 250 °C, respectively. The identification of individual FA methyl esters was achieved by comparing the retention times with commercial standard mixtures (FAME mix 37 components). The conjugated linoleic acid isomers and *trans*- and *cis*-octadecenoic acids were identified with reference to previous reports [28]. FAME were quantified using an internal standard, and nonadecanoic acid (C19:0) methyl ester into each sample prior to methylation. The concentration of FA in the samples was calculated following Le et al. [29]. Each FA content of muscle was expressed as mg/100 g of total FA concent.

#### Quantitative real‑time PCR

Total RNA was extracted from LT muscle (100 mg) using a Trizol reagent (Vazyme, Nanjing, China) according to the manufacturer's instructions. The RNA integrity was assessed using 1% agarose gel electrophoresis. The quality and quantity of RNA samples were determined using a spectrophotometer (DeNovix, USA) at 260 and 280 nm. The RNA samples were converted into the complementary DNA (cDNA) using a reverse transcription kit (BL699A, Biosharp, Hefei, China) according to the manufacturer's instructions. The general reverse transcription system included 1 μg of total RNA, 4 μL of 5 × RT MasterMix, 1 μL of 20 × Oligo dT & Random Primer, and RNase-free H_2_O to a final volume of 20 μL. Quantitative real-time PCR was performed using 2 × Fast qPCR Master Mixture (Green) kit (DiNing, Beijing, China). Primers specific for acetyl-CoA carboxylase (*ACC*), fatty acid synthase (*FAS*), stearoyl-CoA desaturase (*SCD*), sterol regulatory element-binding transcription factor 1(*SREBP-1*), lipoprotein lipase (*LPL*), CCAAT/enhancer binding protein alpha (*C/EBPα*), hormone-sensitive lipase (*HSL*), carnitine palmitoyltransferase-1B (*CPT1B*), peroxisome proliferator-activated receptor γ (*PPARγ*) were designed using Primer 5.0 software (Table 2) and were synthesized by Sangon Biotech Co., Ltd. (Shanghai, China). A portion (1 μL) of each cDNA sample was amplified in a 20 μL PCR reaction, including 0.5 μL of upstream and downstream primers, 10 μL of 2 × Fast qPCR Master Mixture (Green), and ddH_2_O was added to a final volume of 20 μL. The Line-Gene 9600 Plus real-time PCR system (Bioer, Hangzhou, China) was used as follows: 94 °C for 2 min followed by 40 cycles of 94 °C for 15 s, 60 °C for 15 s and 72 °C for 30 s. All samples were assessed in triplicate. The β-actin was selected as the reference gene to normalize mRNA expression of target genes. The relative expression of target genes was evaluated using the 2^–ΔΔCT^ method [30, 31].

**Table 2:** Primers used for quantitative real-time PCR

Genes	Primer sequence (5′→3′)	Product size, bp	GenBank No.
*ACC*	GGCGGGATGGTCTCTTTTC	145	DQ370054.1
	TGGGGACCTTGTCTTCATCATAC		
*FAS*	GGGCTCCACCACCGTGTTCCA	226	NM_001285629.1
	GCTCTGCTGGGCCTGCAGCTG		
*LPL*	GGACACTTGCCACCTCA	278	NM_001285607.1
	CCGCCATCCAGTTCATA		
*SREBP-1*	CTGCTGACCGACATAGAAGACAT	81	NM_001285755.1
	GTAGGGCGGGTCAAACAGG		
*SCD*	TGGCGTTCCAGAATGACGTT	82	NM_001285619.1
	TGGGGATCAGCATCCGTTTC		
*C/EBPα*	CCGTGGACAAGAACAGCAA	141	XM_018062278.1
	GGCGGTCATTGTCACTGG		
*HSL*	CCTCCTCGTGGCTCAACTCCTT	195	XM_018062484.1
	CTGTTGTGTCGCTGCTGTTCCT		
*CPT1B*	GTCTGGGTGATGGGCATCTTCTTC	144	NM_001009259.1
	TCTGGTCAAGTGGCTGGTCTGG		
*PPARγ*	CCTTCACCACCGTTGACTTCT	145	NM_001285658.1
	GATACAGGCTCCACTTTGATTGC		
β-actin	CTCACGGAGCGTGGCTACA	107	JX046106
	GCCATCTCCTGCTCGAAGTC		

*ACC* Acetyl-CoA carboxylase α, *FAS* Fatty acid synthase, *LPL* Lipoprotein lipase, *SREBP-1* Sterol regulatory element-binding transcription factor 1, *SCD* Stearoyl-CoA desaturase, *C/EBPα* CCAAT/enhancer binding protein alpha, *HSL* Hormone-sensitive lipase, *CPT1B* Carnitine palmitoyltransferase-1B, *PPARγ* Peroxisome proliferators activated receptor γ

### Statistical analysis

Data were analyzed using the MIXED procedure (SAS 9.4, SAS Institute Inc., Cary, NC, USA). The model included the fixed effects of CB, RPF and the interaction between CB and RPF, and the random effect of goat. The initial BW was also included in the model as covariate. Tukey’s multiple comparison test was used to examine the significance among treatments when the interaction was significant. Results are reported as least squares means. Effects at *P* < 0.05 were considered statistically significant and effects at 0.05 < *P* ≤ 0.10 as trends.

## Results

### Growth performance and carcass traits

As shown in Table 3, the DMI (*P* = 0.005) and ADG (trend; *P* = 0.058) were increased by supplemented RPF in diets. There was an interaction between CB and RPF for LWBS (*P* = 0.041). The supplementation of CB increased (*P* < 0.05) LWBS when RPF was not added. The HCW was increased either by dietary CB supplementation (trend; *P* = 0.093) or by adding RPF (*P* = 0.046), and similarly the back fat was also increased by supplementation of CB (trend, *P* = 0.100) and RPF (*P* = 0.006). Dietary CB supplementation increased spleen weight (*P* = 0.011) and spleen index (*P* = 0.045) of goats.

**Table 3:** Effects of dietary C*lostridium butyricum* (CB) and rumen protected fat (RPF) on growth performance and carcass traits of goats

Item^1^	CB−		CB+		SEM	*P*-value		
	RPF−	RPF+	RPF−	RPF+		CB	RPF	CB × RPF
Growth performance								
Initial BW, kg	20.7	20.5	21.7	21.7	0.75	0.163	0.865	0.916
Final BW, kg	30.1	31.5	31.0	31.7	1.23	0.552	0.295	0.733
DMI, g/d	1010	1137	982	1095	40.6	0.367	0.005	0.852
ADG, g/d	135.3	158.9	134.3	145.8	8.82	0.430	0.058	0.499
FCR	7.66	7.22	7.54	7.61	0.410	0.733	0.648	0.519
Carcass traits								
LWBS, kg	29.0^b^	31.9^a^	32.1^a^	32.0^a^	1.21	0.030	0.050	0.041
HCW, kg	13.2	14.6	14.5	14.9	0.68	0.093	0.046	0.258
Dressing, %	45.5	45.8	45.1	46.5	1.87	0.759	0.580	0.714
Back fat, mm	2.53	2.77	2.52	2.59	0.066	0.100	0.006	0.980
Organ weight, kg								
Liver	0.46	0.54	0.52	0.52	0.024	0.470	0.151	0.185
Heart	0.12	0.14	0.14	0.13	0.045	0.847	0.346	0.199
Spleen	0.04	0.05	0.06	0.06	0.004	0.011	1.000	0.141
Kidney	0.11	0.12	0.11	0.12	0.010	0.999	0.535	0.754
% of LWBS								
Liver	1.60	1.68	1.61	1.64	0.108	0.877	0.626	0.815
Heart	0.43	0.45	0.43	0.42	0.024	0.530	0.765	0.533
Spleen	0.15	016	0.20	0.18	0.015	0.045	0.699	0.400
Kidney	0.38	0.37	0.35	0.37	0.039	0.617	0.953	0.807

^1^*LWBS* Live weight before slaughtering, *HCW* Hot carcass weight, *FCR* Feed conversion ratio

^a,b^Means within a row with different superscript letters are significantly different (*P* < 0.05)

### Meat quality

The results of meat quality are presented in Table 4. There were interactions between CB and RPF for shear force (*P* = 0.015) and IMF (*P* = 0.049). CB supplementation reduced (*P* < 0.05) shear force in the absence of RPF supplementation. Whereas, the IMF was increased (*P* < 0.05) with combination of CB and RPF supplementation. Furthermore, pH_24h_ (*P* = 0.009) and a* (*P* = 0.007) were increased, and L* (*P* < 0.001) and drip loss (*P* = 0.005) were decreased by supplementing CB. Overall, the supplementation of RPF did not affect the meat quality, except that it reduced (*P* < 0.001) shear force at no CB addition, and increased (*P* < 0.001) IMF regardless of with and without CB.

**Table 4:** Effects of dietary C*lostridium butyricum* (CB) and rumen protected fat (RPF) on meat quality of goats

Item^1^	CB−		CB+		SEM	*P*-vlaue		
	RPF−	RPF+	RPF−	RPF+		CB	RPF	CB × RPF
pH_45min_	6.87	6.82	6.90	7.03	0.092	0.208	0.719	0.347
pH_24h_	5.53	5.59	5.62	5.64	0.025	0.009	0.158	0.411
L* (lightness)	37.7	36.6	34.2	34.6	0.651	< 0.001	0.559	0.253
a* (redness)	11.5	12.3	13.8	12.9	0.509	0.007	0.871	0.122
b* (yellowness)	5.27	5.31	4.94	5.24	0.234	0.395	0.469	0.584
Drip loss, %	4.34	3.89	2.98	3.09	0.345	0.005	0.622	0.434
Cooking loss, %	20.5	25.6	25.0	21.2	2.16	0.989	0.759	0.074
Shear force, N	68.0^a^	51.7^b^	57.4^b^	53.0^b^	2.27	0.050	< 0.001	0.015
Moisture, %	73.2	73.6	72.4	71.4	1.80	0.425	0.859	0.689
Crude protein, %	19.2	19.8	18.6	19.3	0.765	0.478	0.354	0.963
Intramuscular fat, %	4.62^c^	5.51^b^	4.55^c^	5.97^a^	0.159	0.153	< 0.001	0.049

^1^*LT Longissimus thoracis* muscle

^a–c^Means within a row with different superscript letters are significantly different (*P* < 0.05)

### Antioxidative status

The parameters of antioxidants in the LT muscle of goats are shown in Table 5. Dietary CB supplementation increased activities of T-AOC (*P* = 0.050) and GPX (*P* = 0.006) and decreased muscle MDA content (*P* = 0.044). However, the antioxidant activity in the LT muscle was not affected by dietary RPF inclusion.

**Table 5:** Effect of dietary C*lostridium butyricum* (CB) and rumen protected fat (RPF) on antioxidant enzyme activities and MDA content in *Longissimus thoracis* of goats

Item^1^	CB−		CB+		SEM	*P*-value		
	RPF−	RPF+	RPF−	RPF+		CB	RPF	CB × RPF
T-AOC, U/mgprot	0.35	0.37	0.47	0.38	0.031	0.050	0.309	0.139
SOD, U/mgprot	44.9	45.2	44.5	46.0	2.12	0.930	0.684	0.786
CAT, U/mgprot	7.87	8.08	7.90	8.04	0.569	0.997	0.764	0.950
GPX, U/mgprot	122.8	119.2	143.2	132.1	4.56	0.006	0.148	0.437
MDA, nmol/mgprot	2.55	2.49	1.86	2.16	0.150	0.044	0.107	0.065

^1^*T-AOC* Total antioxidative capacity, *SOD* Superoxide dismutase, *CAT* Catalase, *GPX* Glutathione peroxidase, *MDA* Malonaldehyde

### Amino acid composition

As shown in Table 6, an interaction (*P* = 0.003) between CB and RPF was noticed only on the content of lysine (Lys) between CB and RPF; the CB supplementation increased (*P* < 0.05) the content of Lys in the absence of RPF. The contents of essential AA (EAA) (*P* = 0.027), flavor AA (FAA) (*P* = 0.010) and total AA (TAA) (*P* = 0.024) were increased by CB supplementation. The CB supplementation also increased the contents of arginine (Arg) (*P* = 0.013), histidine (His) (*P* = 0.035) and threonine (Thr) (*P* = 0.026) for EAA, and increased non-essential AA (NEAA) contents of serine (Ser) (*P* = 0.018), aspartic acid (Asp) (*P* = 0.023), and glutamic acid (Glu) (*P* = 0.047). Dietary RPF supplementation increased the contents of isoleucine (Ile) (*P* = 0.049) and tyrosine (Tyr) (*P* = 0.044), and decreased (*P* = 0.003) Lys content.

**Table 6:** Effects of dietary C*lostridium butyricum* (CB) and rumen protected fat (RPF) on amino acid composition in *Longissimus thoracis* of goats, % of dry meat weight

Items	CB−		CB+		SEM	*P*-value		
	RPF−	RPF+	RPF−	RPF+		CB	RPF	CB × RPF
Arginine (Arg)	4.52	4.89	5.24	5.04	0.159	0.013	0.587	0.088
Histidine (His)	2.35	2.34	2.55	2.53	0.084	0.035	0.834	0.996
Isoleucine (Ile)	3.26	3.56	3.27	3.32	0.084	0.204	0.049	0.141
Leucine (Leu)	6.04	6.05	6.18	6.41	0.249	0.329	0.637	0.661
Lysine (Lys)	6.37^b^	6.74^b^	8.77^a^	6.52^b^	0.394	0.011	0.003	0.003
Methionine (Met)	1.96	1.90	1.99	1.92	0.063	0.686	0.282	0.874
Phenylalanine (Phe)	2.99	3.10	2.96	3.19	0.110	0.810	0.146	0.570
Threonine (Thr)	3.21	3.23	3.61	3.43	0.126	0.026	0.520	0.449
Valine (Val)	3.59	3.53	3.62	3.62	0.119	0.612	0.832	0.784
EAA^1^	34.3	35.3	38.2	36.0	1.05	0.027	0.544	0.104
Glycine (Gly)	4.04	4.38	4.25	4.44	0.266	0.613	0.336	0.785
Alanine (Ala)	4.95	5.05	4.88	5.54	0.340	0.549	0.280	0.416
Serine (Ser)	2.67	2.68	3.04	2.81	0.096	0.018	0.253	0.230
Proline (Pro)	3.30	3.25	3.03	3.52	0.151	0.972	0.164	0.085
Aspartic acid (Asp)	6.49	6.57	7.38	6.86	0.240	0.023	0.367	0.226
Glutamic acid (Glu)	11.5	11.2	12.1	11.9	0.325	0.047	0.456	0.901
Tyrosine (Tyr)	2.74	3.01	2.41	2.81	0.158	0.109	0.044	0.709
NEAA^2^	35.7	36.1	37.1	37.9	0.835	0.065	0.481	0.830
FAA^3^	31.5	32.1	33.9	33.8	0.722	0.010	0.723	0.648
TAA^4^	70.0	71.4	75.3	73.9	1.59	0.024	0.987	0.369

^1^Essential amino acid = Arg + His + Ile + Leu + Lys + Met + Phe + Thr + Val

^2^Non-essential amino acid = Gly + Ala + Ser + Pro + Asp + Glu + Tyr

^3^Flavor amino acid = Arg + Gly + Ala + Asp + Glu

^4^Total amino acid = EAA + NEAA

^a,b^Means within a row with different superscript letters are significantly different (*P* < 0.05)

### Fatty acid composition

Dietary CB supplementation increased the content of 18:3, n-3 (*P* < 0.001), 20:5, n-3 (*P* = 0.003) and polyunsaturated FA (PUFA) (*P* = 0.048), but it decreased the content of 16:0 (*P* = 0.013) without altering the profiles of other FA (Table 7). In addition, the dietary RPF supplementation increased the content of total FA (*P* = 0.003), 16:0 (*P* < 0.001), *c*9-18:1 (*P* = 0.002), 20:2, n-6 (*P* = 0.014), SFA (*P* = 0.031) and MUFA (*P* = 0.004).

**Table 7:** Effects of dietary C*lostridium butyricum* (CB) and rumen protected fat (RPF) on the fatty acid (FA) composition (mg/100 g of total FA) in *Longissimus thoracis* of goats

Items	CB−		CB+		SEM	*P*-value		
	RPF−	RPF+	RPF−	RPF+		CB	RPF	CB × RPF
Total FA	2,089	2,292	2,138	2,287	51.4	0.678	0.003	0.602
SFA								
12:0	4.6	4.4	4.4	4.7	0.23	0.849	0.810	0.372
14:0	50.6	45.3	50.3	50.4	2.82	0.413	0.370	0.343
15:0	19.6	16.2	21.1	18.6	2.56	0.454	0.261	0.862
16:0	422.5	503.6	385.9	458.6	15.0	0.013	< 0.001	0.782
17:0	40.2	36.7	39.7	43.2	2.17	0.179	0.986	0.119
18:0	333.6	336.8	343.6	343.5	18.7	0.658	0.936	0.930
21:0	12.5	12.6	13.0	12.3	1.16	0.909	0.805	0.742
MUFA								
* c*9-14:1	4.7	4.5	5.1	5.2	0.39	0.193	0.994	0.742
* c*9-16:1	45.6	41.3	46.6	49.5	2.95	0.135	0.820	0.237
* c*9-17:1	29.1	30.6	30.4	31.2	2.68	0.727	0.668	0.906
* c*9-18:1	871.1	992.3	918.4	988.9	26.5	0.418	0.002	0.350
* c*11-18:1	27.3	27.5	26.8	27.9	1.36	0.948	0.647	0.737
* t*10*-*18:1	12.3	12.2	12.5	13.3	0.873	0.472	0.648	0.626
* t*11-18:1	25.2	26.2	24.0	25.5	1.08	0.388	0.235	0.802
PUFA								
18:2, n-6	137.3	138.9	149.3	148.5	9.18	0.253	0.968	0.901
CLA *c*9*t*11	6.89	7.71	6.90	7.44	0.387	0.757	0.094	0.734
18:3, n-3	4.54	5.29	6.78	6.53	0.321	< 0.001	0.441	0.139
20:2, n-6	1.14	1.35	1.15	1.33	0.072	0.990	0.014	0.848
20:3, n-6	3.59	3.91	3.76	3.93	0.132	0.476	0.081	0.572
20:4, n-6	24.8	32.2	31.1	31.0	2.70	0.352	0.189	0.177
20:5, n-3	3.71	4.18	5.20	4.62	0.280	0.003	0.848	0.080
22:5, n-3	6.79	7.35	6.68	6.88	0.507	0.573	0.466	0.725
22:6, n-3	2.82	2.89	2.91	2.97	0.315	0.788	0.834	0.981
SFA	871.0	943.0	844.9	918.9	31.4	0.434	0.031	0.974
MUFA	1027	1147	1076	1154	30.3	0.362	0.004	0.509
PUFA	191.2	203.8	214.8	213.3	7.98	0.048	0.508	0.388

*SFA* Saturated fatty acids, *MUFA* Monounsaturated fatty acids, *PUFA* Polyunsaturated fatty acids

### Lipid-metabolic genes expression

**Fig. 1:**
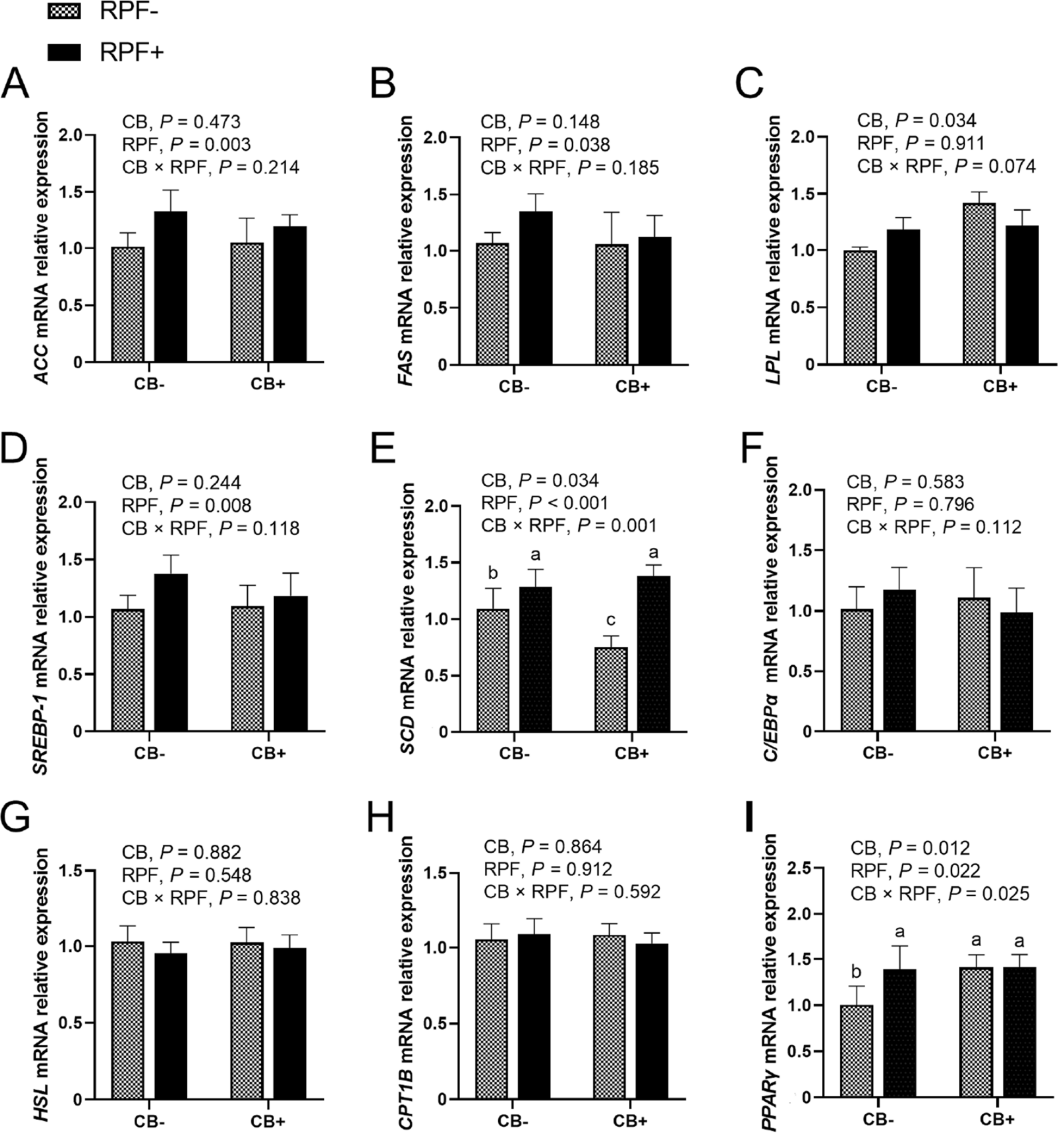
Effects of dietary C*lostridium butyricum* (CB) and rumen protected fat (RPF) on lipid-metabolic genes expression in *Longissimus thoracis* of goats. **A** *ACC* (acetyl-CoA carboxylase); **B** *FAS* (fatty acid synthase); **C** *LPL* (lipoprotein lipase); **D** *SREBP-1*(sterol regulatory element-binding transcription factor 1); **E** *SCD* (stearoyl-CoA desaturase); **F** *C/EBPα* (CCAAT/enhancer binding protein alpha); **G** *HSL* (hormone-sensitive lipase); **H** *CPT1B* (carnitine palmitoyltransferase-1B); **I** *PPARγ* (peroxisome proliferator-activated receptor γ). The mRNA expressions were normalized to β-actin gene expression. All values are expressed as mean ± SEM (*n* = 8). ^a,b^ Means within a row with different superscript letters are significantly different (*P* < 0.05)

As shown in Fig. 1, the interactions between CB and RPF were observed for *SCD* (*P* = 0.001) and *PPARγ* (*P* = 0.025). Dietary CB supplementation did not change the expression of *SCD* when it was combined with RPF, but it downregulated (*P* < 0.05) the expression of *SCD* without RPF supplementation. Dietary CB supplementation upregulated (*P* < 0.05) the expression of *PPARγ* when RPF was not added in diets. Moreover, supplementation of CB upregulated (*P* = 0.034) the *LPL* expression in LT muscle. Dietary RPF supplementation upregulated the expression of *ACC* (*P* = 0.003), *FAS* (*P* = 0.038), and *SREBP-1* (*P* = 0.008).

## Discussion

### Growth performance and carcass traits

To date, the information regarding the effect of CB supplementation on the growth performance of ruminants is still scarce. Li et al. [32] observed that the growth performance of Holstein heifers was improved by dietary CB inclusion. However, Zhang et al. [10] reported that supplementation of CB in goat diet did not change the fattening performance of goats, which is consistent with the present study. Moreover, Cai et al. [11] found that supplementation of CB alone or combined with *Saccharomyces cerevisiae* both improved growth performance of heat-stressed goats. Thus, it can be suggested that the beneficial effects of CB supplementation on animal growth performance varied with studies due to the animal species used (dairy vs. goats) or environmental conditions (e.g., heat stress). It has been reported that the amount and composition of fat supplemented in diets are the key factor to affect the DMI of ruminants [33–35]. In the present study, supplementation of RPF increased DMI, which is consistent with the study reported by De Souza et al. [36] who found an increase of DMI of dairy cows fed an C16:0-enriched diet. Similarly, Bai et al. [35] reported that supplemental rumen bypass fat (87% C16:0 + 10% C18:0) increased DMI of Angus bulls. The possible explanation could be the C18:0 content (5%) in the RPF, as speculated by Rico et al. [37] that C18:0 inhibit the secretion of hypophagic gut peptides, such as glucagon-like peptide 1 and cholecystokinin which would limit a sense of fullness, consequently allowed for a greater DMI. In addition, previous studies reported that supplementation of RPF increased the nutrient digestibility such as protein, lipid or fiber in steers or ewes [38, 39]. Therefore, the greater ADG due to RPF supplementation could be explained by the greater DMI and nutrient digestibility.

In this study, CB supplementation increased the LWBS, but it was interacted with RPF, the effect of either CB or RPF on the increased LWBS appeared to be independent, rather than additive. The trend of increase of HCW by CB is consistent with the increased LWBS. The increase in LWBS with CB in this study may be due to the fact that the CB can provide metabolites, especially short-chain FA, as an energy source to the digestive enzyme system, thus increase the BW [15]. The spleen is the largest immune organ in animal body, which can improve the body resistibility and minimize the pathogenic bacteria from invading the body, therefore its development is believed to be directly associated with the immune function of the body. The increased spleen weight and its percentage of LWBS, suggesting that feeding CB to goats may potentially improve the immune function.

Dietary energy level is closely associated with carcass traits, and the dressing percentage is considered as a key indicator for measuring carcass traits. In this study, the increased HCW with adding RPF was in agreement with the increased LWBS, while, the increased backfat thickness by RPF supplementation could be explained by increasing fat deposition as a result of adding FA from RPF. The increased LWBS, HCW and backfat thickness without affecting the dressing percentage with RPF addition were in agreement with Awawdeh et al. [40], who reported dietary fat supplementation improved LW and backfat thickness of lambs without changing the dressing percentage. It speculates that adding dietary fat would be especially toward to backfat deposition with minimum impact on dressing.

### Meat quality

Muscle pH is primarily related to its shearing force, water retention capacity and meat color. Glycolysis is a major metabolic pathway in animal postmortem period, resulting in accumulation of lactic acid, which leads to a rapid decline in muscle pH [41]. In this study, although the pH_45min_ was not changed, the increased pH_24h_ by adding CB suggest a reduction of muscle glycolysis rate. Notably, drip loss of meat is related to the ultimate pH and the speed of pH drop, the lower drip loss as a result of raised pH, as reported by Di Luca et al. [42]. Results from the current study demonstrated that the addition of CB decreased drip loss, which is in accordance to the results of Liu et al. [12]. Studies have reported that the antioxidant capacity of meat is inversely correlated with drip loss, and the damage of cell membrane integrity caused by lipid oxidation is associated with increased drip loss [43]. Therefore, the decrease of drip loss in our study indirectly reflects the inhibitory effect of CB on oxidative damage and exerting positive effects on the water-holding capacity of muscle.

Meat color is one of most used criteria in assessing meat quality and is the single most important driving factor in a consumer's decision to purchase meat [44]. The meat color mainly depends on oxidation and light reflection. Consumers prefer bright red meat because they associate to a red color with freshness. The content of myoglobin is closely related to meat color values, when oxymyoglobin is oxidized to methemoglobin, the color of meat changes from bright red to reddish brown [45]. Studies have found that exogenous antioxidants supplementation could prevents lipid oxidation, thus stabilizing oxymyoglobin content [46]. In this study, CB supplementation would improve the oxidative stability due to the increased activities of T-AOC and GPX, and decreased the content of MDA in LT muscle, which was reflected to meat color changes with an increase in a* values and a decrease in L* values. In a similar study, Cai et al. [11] found that dietary addition with CB increased the a* value and decreased the L* value in muscle of pigs. Tenderness has been considered as the most important palatability characteristic of meat, which can be evaluated by shear force. In this study, it was found that supplementation of CB decreased shear force of LT muscle. This may be attributed to the decrease of drip loss, which is positively correlated with meat tenderness. In addition, the decrease in shear force when goats were fed RPF in the present study can be attributed to the higher IMF, as reported in beef cattle [47]. However, although the RPF had the influence on shear force, it had no effect on pH value, meat color and water retention capacity in the present study. In support of our findings, Parente et al. [19] showed that diets supplemented with oil containing a variety of mixed FA decreased shear force, but had no effect on pH, meat color and other meat physical traits.

The IMF positively influences sensory quality traits of meat including tenderness, juiciness, and flavor of meats [48]. In this study, although the supplementation of CB alone did not change IMF content, it increased the IMF content with RPF addition in the diets, suggesting an additive effect between CB and RPF on the IMF content. We speculate that adding CB in the diet may facilitate the deposition of FA in the muscle when a high dietary FA content is available.

### Oxidative stability of the *longissimus thoracis* muscle

Lipid oxidation has a negative impact on meat quality and shelf life, which can lead to deterioration of flavor, color and nutritional value of meat [49]. Reducing lipid peroxidation or improving antioxidant status is an effective way to increase the quality and shelf life of meat products. It is reported that CB supplementation in broilers’ diets increased SOD activitiy in liver tissues while decreasing MDA concentrations in serum and liver tissues [6]. Our findings, as first demonstrated, that dietary CB supplementation in finishing goats beneficially increased T-AOC and GPX activities and decreased lipid oxidation products MDA in LT muscle. The antioxidant activity of muscle was also closely related to meat quality. Zhang et al. [50] reported that increased antioxidant activity in meat can inhibit oxidative stress, maintain meat color stability and reduce drip loss. Therefore, the current results suggested that supplementation of CB has a positive effect on regulating the redox state of goat’s LT muscle and improving meat quality. The effect of CB on antioxidant capacity may be partly attributed to the beneficial effect of butyric acid and H_2_ produced by its metabolism. Butyric acid can regulate oxidative damage by reducing reactive oxygen species and increase antioxidant enzyme levels [51], and H_2_ mediates selective scavenging of harmful substances such as free hydroxyl radicals and oxygen free radicals [52].

### Amino acid profile of *longissimus thoracis* muscle

The flavor and nutritional value of meat are closely related to the profiles and content of AA. Specific AA are thought to be important in contributing to its desired flavors such as Arg and Phe provide a bitter taste; Glu and Asp show an umami flavor; Gly, Ala, and Ser present a sweet taste [43]. The EAA are essential to meet the human AA requirements and play critical role in growth, regulating immune function and maintaining normal metabolism. In the present study, dietary CB supplementation increased the concentrations of EAA (+6.5%), FAA (+6.5%) and TAA (+5.5%), and the concentrations of individual AA (from +6.1% to +16.6%) including Arg, His, Lys, Thr, Ser, Asp and Glu. It is worth mentioning that Arg, Asp and Glu belong to the FAA, and the Glu is the most important FAA, which plays an important role in the freshness of meat and buffering sour and salty taste. Therefore, our results suggested that supplementation of CB could improve the flavor and nutritional value of goats’ meat. In contrast, dietary RPF supplementation appeared to have limited effect on AA profiles and its contenet in meat. The increased the content of Ile and Tyr may have been due to improved digestibilities of AA as reported that dietary fat can improve AA digestibility [53]. However, the reduced Lys content by RPF in combination with CB addition is not clear, and it necessitate further research to understand the mechanism by which RPF influences the AA composition.

### Fatty acid profile of *longissimus thoracis* muscle

It is well documented that FA are important indicators to evaluate meat quality and nutritional value, as well as the basis of the characteristic flavor of meat. In recent years, researchers have increasingly focused on the regulation of FA profiles in meat as cardiovascular heart disease are considered closely related to dietary with high SFA, specifically C16:0 and myristic acid (C14:0) [54]. The MUFA and PUFA play important roles in protecting the heart, lowering blood lipids and regulating blood sugar. Therefore, a decrease in the SFA content and an increase in the UFA content can improve the nutritional value of goats’ meat. The current findings showed that CB supplementation decreased the content of C16:0, but increased that of C18:3, C20:5n-3 and PUFA in the LT muscle. The α-linolenic acid (C18:3n-3) is known as a precursor for the synthesis of EPA (C20:5n-3) and DHA (C22:6n-3), which are converted to EPA and DHA via elongation and desaturation enzymes located in the liver. EPA and DHA play important regulatory roles in human health, which can prevent the synthesis of lipoproteins in the liver, improve cardiovascular function and regulate inflammatory immune function of the body. Thus, it can be inferred from the above studies that CB has the potential to improve FA profile of muscle and enhance the nutritional value and flavor of mutton. The mechanism by which CB regulates muscle FA composition and content of muscle PUFA is unclear. We speculate that butyric acid, a metabolic product of CB, may play a key role in maintaining intestinal health, regulating lipid metabolism and epithelial barrier function, which may benefit digestion and absorption processes of PUFA in the gastrointestinal tract. In addition, studies have suggested that the increased PUFA concentrations in meat may be due to the protective effects of antioxidants in the diet [55], which is consistent with the increased muscle antioxidant capacity by CB in the present study.

Manipulating diet is an effective way to affect the FA composition in ruminant meat. The oleic acid (*c*9-18:1) is reported to be the most abundant UFA in mutton, and has an effect on lowering cholesterol and regulating blood lipid [36]. The higher proportions of *c*9-18:1 and C16:0 in LT muscle with RPF supplementation in the current study may be explained by the large amount of C16:0 and C18:1 supplied by the RPF. These findings are consistent with Ladeira et al. [56], who reported that dietary supplementation with RPF increased the concentrations of C16:0, C18:1 and MUFA in the LT muscle of bulls.

### Relative mRNA expression in *longissimus thoracis* muscle

Muscle lipid accumulation is generally the result of a balance between lipid availability (via circulatory lipid uptake or de novo lipogenesis) and lipid disposal (via FA oxidation). The process of lipid accumulation involves many key enzymes and transcription factors [35]. *LPL* is a rate limiting enzyme used to hydrolyze lipoproteins, chyle granules and low-density lipoproteins. *LPL* catalyzed reaction products, fatty acids and monoglycerides, are partially absorbed by adipose tissue and skeletal muscle and stored in the form of neutral lipids. In this study, CB addition increased expression of *LPL*. It is reported that overexpression of *LPL* is related to increased triacylglycerol accumulation and fat deposition in mammalian muscles [57]. Previous studies have reported that the expression of *SCD* is negatively correlated with PUFA, EPA and DHA in beef cattle [58], which is consistent with our results that the expression of *SCD* in the LT muscle of goats was decreased, while the content of PUFA and EPA was increased by dietary CB supplementation. Furthermore, *SCD* can catalyze the dehydrogenation of SFA to MUFA, especially catalyzes C16:0 and C18:0 into C16:1 and C18:1, respectively, and is closely related to the differentiation of preadipocytes. In this study, we found increased expression of *SCD* when goats were fed RPF diets, which may be the reason that RPF increased the content of C18:1 in LT muscle, indicating that RPF could change the expression of *SCD*, thereby affecting the composition of FA in muscle tissue.

The *PPARγ*, as a member of the nuclear receptor superfamily, can induce adipocyte differentiation as well as regulate the expression of *ACC*, *FASN* and *LPL* to induce the accumulation of lipid droplets in skeletal muscle, thereby increasing the content of IMF [59]. In our study, dietary CB supplementation increased the expression of *PPARγ* indicating that CB could promote fat deposition. The increased expression of *PPARγ* may be partly related to butyric acid produced by the metabolism of CB. It has been reported that butyric acid could affect lipogenesis by regulating the PPARγ signaling pathway [60]. In addition, the mRNA expression of *PPARγ* was also increased by RPF in LT muscle in this study. Similarly, Li et al. [61] found that oleic acid increased the mRNA expression of *PPARγ* in bovine muscle satellite cells. The *SREBP-1* is a nuclear transcription regulator that regulates the expression of many downstream target genes involved in lipid metabolism. *ACC* is the rate-limiting enzyme in de novo synthesis of FA, catalyzing the synthesis of malonyl-CoA for subsequent biosynthesis of long chain FA. *FAS* is considered to be a determinant of the maximal capacity of a tissue to synthesize fatty acids by the de novo pathways, which plays a catalytic role in the last step of FA biosynthesis pathway. Previous studies have reported that the expressions of *ACC* and *FAS*, in LT muscle of Korean steers are positively correlated with IMF content [62]. It can be seen from our study that dietary addition of RPF increased the expressions of *ACC*, *FAS* and *SREBP-1* in LT muscle, indicating that RPF could contribute to fat synthesis. Yang et al. [63] reported that the expressions of *ACC* and *FAS* in the LT muscle were enhanced with increasing dietary energy levels. RPF can improve the energy density of the diet, and high dietary energy means that the cells can absorb more energy, thus increasing the expression of these fat-producing genes, promoting lipid metabolism, and resulting in fat deposition in tissue.

## Conclusion

In conclusion, the CB supplementation in the goat diet improved meat quality by enhancing the antioxidant capacity, color and pH, and improving the AA and FA composition of LT muscle. Specifically, dietary CB supplementation increased the IMF content, when the RPF was supplemented in the diet. It suggests an additive effect between CB and RPF on improving the meat quality and composition. In addition, the RPF supplementation in goat diet demonstrated an improvement of the growth performance, carcass traits, and FA profiles by increasing 16:0 and *c*918:1 concentration. The expressions of *ACC*, *FAS*, *SREBP-1* and *PPARγ* were also increased by RPF supplementation, and consequently increased the intramuscular fat content. It concludes that supplementation of goat diet with CB and RPF has beneficial effect on improving the carcass traits, meat quality, and promoting fat deposition by upregulating the expression of lipogenic genes of LT muscle.

## Data Availability

The datasets produced and/or analyzed during the current study are available from the corresponding author on reasonable request.

## Abbreviations

AA: Amino acid
ACC: Acetyl-CoA carboxylase α
BW: Body weight
CAT: Catalase
CB: *Clostridium butyricum*
C/EBPα: CCAAT/enhancer binding protein alpha
CPT1B: Carnitine palmitoyltransferase-1B
FA: Fatty acids
FAME: Fatty acid methyl esters
FAS: Fatty acid synthase
GPX: Glutathione peroxidase
HCW: Hot carcass weight
HSL: Hormone-sensitive lipase
IMF: Intramuscular fat
LPL: Lipoprotein lipase
LT: *Longissimus thoracis*
LWBS: Live weight before slaughter
MDA: Malondialdehyde
MUFA: Monounsaturated fatty acids
PPARγ: Peroxisome proliferators activated receptor γ
PUFA: Polyunsaturated fatty acids
RPF: Rumen protected fat
SCD: Stearoyl-CoA desaturase
SFA: Saturated fatty acids
SOD: Superoxide dismutase
SREBP-1: Sterol regulatory element-binding transcription factor 1
T-AOC: Total antioxidant capacity
